# Rheumatic Heart Disease is Missing from the Global Health Agenda

**DOI:** 10.5334/aogh.3426

**Published:** 2021-11-17

**Authors:** Wubishet Belay, Muktar H. Aliyu

**Affiliations:** 1 Monroe Carell Jr. Children’s Hospital at Vanderbilt, Nashville, TN, US; 2 Vanderbilt Institute for Global Health (VIGH), Nashville, TN, US

## Abstract

Rheumatic heart disease (RHD) is a complication of untreated throat infection by Group A beta-hemolytic streptococcus with a high prevalence among socioeconomically disadvantaged populations. Despite its high incidence and prevalence, RHD prevention is not a priority in major global health discussions. The reasons for the apparent neglect are multifactorial, including underestimated morbidity and mortality burden, underappreciated economic burden, lack of public awareness, and lack of sustainable investment. In this review, we recommend multisectoral collaboration to tackle the burden of RHD by engaging the public, health experts, and policymakers; augmenting funding for clinical care; improving distribution channels for prophylaxis, and increasing research and innovation as critical interventions to save millions of people from preventable morbidity and mortality.

## Background

Acute rheumatic fever (ARF) and rheumatic heart disease (RHD) are complications of untreated throat infection by Group A Streptococcus GAS [1]. ARF and RHD are common in children aged 5–15 years and cause multivalvular heart disease with progressive valvar dysfunction and untimely death if progression is not prevented or the lesions are not corrected by surgery [2]. Primary (treating GAS pharyngitis) and secondary (preventing ARF recurrence in a patient with RHD) preventive measures are available and cost-effective [3,4]. As a result, ARF and RHD are rare in the developed world [5]. But the story is different in low- and middle-income countries (LMICs) and some developed countries, where the prevalence of RHD continues to be high, especially among socioeconomically disadvantaged populations [2,6,7,8].

Despite the high incidence and prevalence of RHD in under-resourced settings, the disease is missing from major global health discussions [9,10]. There is a lack of urgency from responsible bodies with power and resources [11]. Cutting-edge research and innovations are lacking. There is also not enough funding for research compared to the disease burden, which likely contributes to the lack of progress in averting this public health crisis in developing nations [9,12]. This article discusses the possible reasons why RHD continues to be neglected in the global health world.

## Underestimated Epidemiologic burden

Despite remarkable success in the developed world, ARF and RHD continued to contribute significantly to chronic disease burden in Southeast Asia and Sub-Saharan Africa [13]. Echocardiography-based screening has increased the detection rate of latent RHD in young children and adults [14]. Surveys conducted in various countries showed echocardiographic-based RHD screening detects a significant additional number of children with latent RHD, estimated to be from 7.5–51.6 per 1000 children [6,13,15].

The lack of widespread use of echocardiographic screening in many LMICs has considerably underestimated the prevalence, incidence, morbidity, and mortality burden of RHD [16].

## Underappreciated mortality burden

RHD is the most frequent cause of heart failure in sub-Saharan Africa, with a short-term mortality rate of close to 18% in children and young adults [17]. The lack of access and high cost of cardiac surgery in LMICs has limited the availability of surgical treatment to most RHD patients [18]. Interestingly, RHD has a mortality rate comparable to that of rotavirus and about 50% of that of malaria [19,20]. Compared to the level of resource support that rotavirus and malaria programs enjoy, funding for RHD programs are not even close to comparison [21]. Importantly, most of the deaths associated with RHD occur in the teenage years and young adulthood and are therefore not picked up by politically sensitive measures like under five mortality [22]. This circumstance again has contributed to the lack of urgency from a research and disease control point of view.

## Underestimated disability burden

RHD is a known cause of significant disability burden in affected individuals. Patients with multivalvular heart disease often are limited in their exercise capacity [23]. In rural sub-Saharan Africa, where most people are farmers, it is nearly impossible to be productive with RHD [24]. These patients will ultimately require frequent medical attention, resulting in a productive member of the family staying home and caring for them [25]. A Global Burden of Disease Study highlighted that the rate of disability-adjusted life years due to RHD was estimated to be 142.6 per 100,000 population in 2015. The highest age-adjusted rates are found in Southern Asia, Oceania, and Africa [26].

Unlike other diseases like Malaria and HIV, where the effect of the illness on productivity is acutely observed, RHD usually causes progressive loss of productivity over the years, as the clinical course spans years to decades [27].

## High economic burden

The economic burden of RHD in LMICs is the least studied aspect of this issue, with few studies on this over the last three decades [28]. A study from Fiji showed a high cost of illness due to RHD [27]. Another study showed that RHD causes massive economic effects worldwide due to the premature death of young and working-age adults. It is estimated to cause a 2.2 trillion-dollar loss worldwide annually [29].

We believe that the underappreciated economic impact and the lack of return for the investment are the main reasons RHD is an underfunded, underappreciated, and neglected disease of the poor and the voiceless. The burden of RHD does not attract as much attention as other chronic diseases, as it usually does not cause acute mortality. While these attributes of the disease make it expensive from an access point of view and definitive treatment, the lack of advocacy from the global health community to promote prevention while a cheaper option exists puts a ‘dead fly’ in the global health community’s ointment.

## Lack of public awareness

The public has an essential role to play in RHD prevention and control [30]. Public awareness of RHD is another public health issue that is not well addressed. Many patients do not know the relationship between a sore throat and rheumatic fever and its consequences [31]. Multicenter data on public awareness of ARF and RHD are lacking currently. More studies are needed to identify knowledge gaps to help design public awareness methods in areas with a high disease burden.

## Investing in ARF and RHD: State of the art cardiac center versus prevention?

At a 2015 meeting in Addis Ababa, Ethiopia, experts issued seven priority areas to reduce RHD-related mortality by 25% by 2025 and eliminate ARF and eradicate RHD. While this is a good first step, there is not enough evidence to suggest if the seven priority areas are embraced by policymakers, funding agencies, and public health experts [32]. We believe the challenge in ARF and RHD treatment, prevention, and control starts here. While this action taken at the African Union level will serve as a primer for future efforts, without a genuine commitment to this issue, recommendations from experts alone will not result in a measurable difference in the prospects of eradicating this disease.

There is a growing interest in state-of-the-art cardiac care in many countries. However, investment in advanced care should not hamper effective and very cheap preventive activities, such as treating GAS pharyngitis and strengthening secondary prevention efforts. Investment in RHD should be focused on where the return is the highest, which is prevention. Fortunately, Benzathine penicillin (BPG) is an effective antibiotic for primary and secondary prophylaxis. It is cost-effective and works well. We believe it is worth investing in BPG drug distribution chains and health systems to make it available in many LMICs.

Another point to consider when planning and comparing the cost of investment between prevention and definitive treatment is the cost of treatment programs. RHD surgery is not completely curative. Patients will require expensive medical care and follow-up after surgery, while prevention averts the issue altogether. For countries with limited Financial resources, investing in primary and secondary prevention should be an attractive option.

## Can RHD policymakers learn from the success of HIV programs?

HIV came close to wiping out the most productive population of sub-Saharan Africa while its leaders were in denial [33]. There is a similar attitude to rheumatic heart disease. The HIV crisis was addressed after it cost Africa its productive society and massive economic loss. The story of RHD is similar to the story of HIV three decades ago. HIV needed multinational global cooperation and push from global health stakeholders to control the epidemic. The HIV case study can be a model for a disease affecting the poor that keeps on being neglected by lawmakers and global funders.

Although RHD does not cause a threat to the developed world as much as HIV does, the economic impact of morbidity and mortality is not far from HIV. While one African patient with HIV can get lifesaving drugs for 75 dollars a year, a single cardiac surgery for RHD costs significantly more when it is available [34,35]. The global health community worked tirelessly to lower the cost of HIV drugs and discover newer and safer medications. A similar effort is essential to avert this crisis.

In addition to the expert guidance from the Addis Ababa meeting, the World Health Assembly (WHA) adopted a resolution dedicated to RHD in 2018. By advocating for an organized global response to RHD, we hope that this resolution will significantly boost national prevention efforts [36].

RHD prevention has few options from a pharmacologic point of view. BPG is a very painful injection. Currently, there are no ongoing studies to come up with newer and more reliable options. The global health community should rally behind efforts to improve the prophylactic options for RHD.

## Conclusion

RHD continues to be a significant public health problem in many LMICs. The prevention and treatment programs are underfunded, and its public health burden is underestimated. All rounded appreciation of the disease burden is essential. Social determinants of health such as healthcare access, housing, and household income are crucial in preventing diseases of poverty like rheumatic heart disease. Multisectoral collaboration by engaging the public, health experts, and policymakers is therefore critical. Augmenting funding for prophylaxis distribution and research and innovation is crucial to saving millions of people from preventable morbidity and mortality.
